# Two novel *MYH7* proline substitutions cause Laing Distal Myopathy-like phenotypes with variable expressivity and neck extensor contracture

**DOI:** 10.1186/s12881-016-0315-1

**Published:** 2016-08-12

**Authors:** Miora Feinstein-Linial, Massimo Buvoli, Ada Buvoli, Menachem Sadeh, Ron Dabby, Rachel Straussberg, Ilan Shelef, Daniel Dayan, Leslie Anne Leinwand, Ohad S. Birk

**Affiliations:** 1 The Morris Kahn Laboratory of Human Genetics at the National Institute of Biotechnology in the Negev and Faculty of Health Sciences, Ben Gurion University, Beer Sheva, 84105 Israel; 2 Department of Molecular, Cellular and Developmental Biology, University of Colorado, Boulder, CO 80309-0347 USA; 3 Department of Neurology, Edith Wolfson Medical Center, Holon, Israel; 4 Affiliated to Sackler Faculty of Medicine, Tel Aviv University, Ramat Aviv, Tel Aviv, Israel; 5 Neurology Institute, Schneider Children’s Medical Center, Petah Tikvah, Israel; 6 Diagnostic Imaging Institute, Soroka Medical Center, Faculty of Health Sciences, Ben Gurion University, Beer-Sheva, 84101 Israel; 7 Genetics Institute, Soroka Medical Center, Faculty of Health Sciences, Ben-Gurion University of the Negev, Beer-Sheva, 84101 Israel

**Keywords:** MYH7, Laing distal myopathy, Proline mutations, Myosin rod

## Abstract

**Background:**

Human skeletal muscles express three major myosin heavy chain (MyHC) isoforms: MyHCIIx (MYH1) in fast type 2B muscle fibers, MyHCIIa (MYH2) in fast type 2A fibers and MyHCI/β-cardiac MyHC (MYH7) in slow type I skeletal fibers and cardiac ventricles. In line with its expression pattern, *MYH7* mutations have been reported in association with hypertrophic or dilated cardiomyopathy, skeletal myopathies or a combination of both. We analyzed the clinical and molecular phenotype of two unrelated families of Jewish Moroccan ancestry that presented with apparently autosomal dominant inheritance of progressive Laing-like distal myopathy with non-specific myopathic changes, but uncommon marked contractures and wasting of the neck extensors.

**Methods:**

Clinical phenotyping, whole exome sequencing and restriction analysis, generation of mutants followed by cell culture transfection and imaging.

**Results:**

Using whole exome sequencing we identified in both families two novel heterozygous proline substitutions located in exon 31 of *MYH7* within its rod domain: c.4309G>C (p.Ala1437Pro) and c.4301G>C (p.Arg1434Pro). Here we show that the phenotype caused by these mutations includes marked cervical muscle contracture, and report that the severity of the phenotype varies significantly, to the extent of non-penetrance in one of the families. Finally, we provide evidence that both proline substitutions impair myosin self-assembly in non-muscle cells transfected with β-myosin constructs carrying the mutations, but do not prevent incorporation of the mutant molecules into the sarcomere.

**Conclusions:**

This study expands our clinical and molecular knowledge of *MYH7* rod mutations causing skeletal myopathies, and underscores the importance of discussing disease penetrance during genetic counseling.

## Background

Muscle contraction depends on the intrinsic contractile properties of cardiac and skeletal myocytes, and is driven by myosin, the major motor protein of both cardiac and skeletal muscles [1]. Myosin consists of an amino-terminal motor/head domain and a carboxy-terminal tail or rod domain. While the ATP-hydrolyzing motor domain generates force, the long coiled-coil rod directs myosin assembly into the thick filament [2]. Different skeletal myofibers express specific compositions of myosin heavy chains; for instance, *MYH7,* encoding the slow/β-cardiac MyHC, is the predominant motor isoform found in slow twitch type I fibers, which display oxidative metabolism and high endurance. This myosin is also the major isoform in human cardiac muscle [3].

More than 350 *MYH7* mutations have been associated with different clinical phenotypes: hypertrophic and dilated cardiomyopathy (MIM192600), non-compaction and restrictive cardiomyopathy (MIM613426), Ebstein anomaly (MIM224700), Laing distal myopathy (MPD1; MIM160500), myosin storage myopathy (MIM608358), and scapuloperoneal syndrome, myopathic type (MIM181430). Although most of these mutations have dominant negative activity, homozygous-recessive or compound heterozygous patients have also been identified [4, 5].

The clinical spectrum of skeletal myopathies associated with *MYH7* mutations is variable but is always associated with muscle weakness. Laing distal myopathy (LDM) muscle biopsies sometimes show i) changes in muscle fiber size with type I hypotrophy, ii) co-expression of slow and fast myosin, iii) mild necrosis and regeneration iv) while mitochondrial abnormalities are not a common feature of LDM biopsies, the presence of serpiginous cytoplasmic bodies, possibly as sign of secondary sarcomeric disruption, had been described in one family with a substitution to proline [6, 7] In contrast, myosin storage myopathy muscle biopsies consistently show eosinophilic subsarcolemmal aggregates [8]. In this study, we identified in two families with a LDM-like phenotype two novel proline missense mutations located in the rod domain of the slow/β-cardiac MyHC. In addition to muscle weakness, these mutations are also associated with marked cervical muscle contractures; interestingly, one of the families shows partial penetrance. Both proline substitutions interfere with myosin’s intrinsic ability to self-assemble into spindle-shape ordered structures in non-muscle cells. Nevertheless, these mutations do not hinder proper incorporation of myosin into the thick filaments of cultured cardiomyocytes.

## Methods

### Patients

**Fig. 1:**
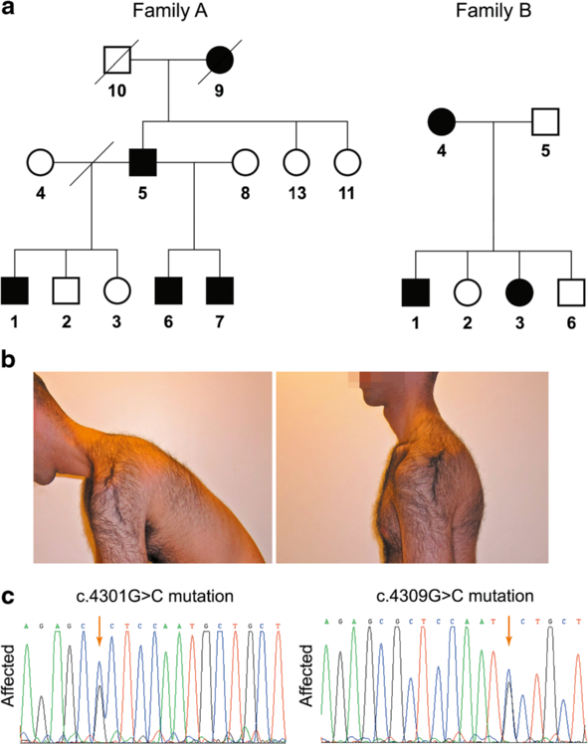
Pedigrees and mutations. **a** Two unrelated families (A, B) of Jewish Moroccan origin presenting with a unique phenotype of skeletal myopathy. **b** Severe contracture of the neck extensors and dorsal para-spinal muscles, prohibiting flexion of the neck (individual 6, family A, 28 year old male, in maximal neck flexion). **c** Sanger sequencing demonstrating the two novel *MYH7* rod domain proline substitution mutations c.4309G>C (p.Ala1437Pro) and c.4301G>C (p.Arg1434Pro) in affected individual 6 of family A and individual 2 of family B, respectively

Seven affected individuals of two apparently unrelated non-consanguineous families of Jewish Moroccan ancestry were studied (Fig. 1a). Clinical phenotyping was determined by an experienced neurologist and geneticist.

### Muscle biopsies

Muscle biopsies were taken from the quadriceps of patient A5 and B4 (Fig. 1a). Part of the muscle was fixed with formalin and embedded in paraffin. Another sample was frozen in isopentane chilled in liquid nitrogen for biochemical studies. Transverse sections (7 μM thick) were stained with haematoxylin and eosin, modified Gomori trichrome, Periodic acid–Schiff, Oil red O, NADH-tetrazolium reductase, succinate dehydrogenase and cytochrome oxidase, ATPase at pH 9.4 and after preincubation at pH 4.3 and 4.6. Immunohistochemistry studies included dystrophin 1-3, sarcoglycans, merosin, caveolin 3, lamin A/C and dysferlin.

### Magnetic resonance Imaging (MRI)

MRI was performed with a 3Tesla MRI system (Ingenia Philips). The following pulse system was used: T1-weighted images with repetition time (TR) of 600 ms and an echo time (TE) of 16 ms. Slices 4 mm thick were taken.

### Sequencing

Whole exome sequencing (HiSeq2000, Illumina, San Diego, CA) was done using paired-end (2 × 100) protocol at a mean coverage of 30-fold (85 %–90 % of all exonic nucleotides were covered by >10 reads) as previously described [9]. For exome enrichment, we used NimbleGen SeqCap EZ Human Exome Library v2.0 (Roche NimbleGen, Madison, WI) targeting 44.1 Mb regions. Sequencing read alignment, variant calling and annotation were performed by DNAnexus (DNAnexus Inc., Mountain View, CA; dnanexus.com).

### Restriction analysis

PCR primers were designed generating a recognition site for MowI that is abolished by the c.4309G>C mutation (Primers: TTAATGCCAAGTGCTCCTCG, aaaaaaaAGTTCCTCTGCTTCTTGTCCAGGtC). Wildtype (WT) allele restriction products are 96 and 37 bp, versus uncut 133 bp for the mutant allele. The same primers were used for c.4301G>C mutation screening, where HaeII recognition site exists only in the WT sequence. WT allele restriction products: 83 and 50 bp, mutant products is 133 bp.

### DNA constructs

Isolation of the mouse β-myosin cDNA was carried out by RT-PCR as follows: total RNA was isolated from C57BL/6 J mouse soleus muscle with TRI Reagent. cDNA was prepared using 2 μg of RNA, random primers d(N6) and SuperScript III. PCR reaction was performed with MYH7 - specific primers Beta F (ATGGCGGATGCAGAGATGGCTG) and Beta R (CTCCTCATTCAGGCCCTTGCAC-) and iProof High Fidelity DNA Polymerase. The 5.8Kb PCR product, corresponding to the full-length β-myosin gene, was then cloned into the pEGFP-C2 plasmid as EGFP carboxy-terminal fusion according to standard procedures. In this construct, the GFP and myosin genes are separated by a 13 amino acid linker derived from the plasmid multi-cloning site (spanning from the EagI and the EcoRI restriction sites). The β-myosin EGFP mutants carrying the two mutations, R1434P and A1437P, were generated by inverse PCR [10] with the following primers: R1434P, forward and reverse primers respectively:

CCTCCAATGCCGCCGCC; GCTCCACGTCCACCATCAGGTC; A1437P: forward and reverse primers respectively: CCCGCCGCCGCAGC; ATTGGAGCGCTCCACGTCCAC.

### Cell culture transfection and imaging

COS-7 cells and neonatal rat ventricular myocytes (NRVMs) were cultured, transfected and imaged as previously described [11].

## Results

### Clinical phenotype

All affected individuals of family B and some members of family A reported difficulties running and taking part in sport activities in childhood. They had difficulties in dorsiflexion of the feet and wasting of the thighs, which in some patients at later ages culminated in difficulty getting up from a chair or climbing stairs without a railing. Detailed physical examination is given below for five patients, including the probands (individuals 6 of family A and B3 of family B).

Family A: Male individuals 5 (70 year old) and 6 (28 year old) had very similar phenotypes: both had frontal balding, as well as severe contracture of the neck extensors and dorsal para-spinal muscles, preventing passive flexion of the neck beyond the neutral position (Fig. 1b). Contractures of the back flexion were also found: during bending forward the spine remained rigid. Patient 6 had mild proximal weakness of the upper and lower limbs, moderate weakness of feet dorsiflexors and mild contractures of the elbows. The 70-year-old patient 5 had more progressive disease, with weak proximal upper limbs muscles, especially the deltoids and pectoralis (4/5 on the Medical Research Council scale) and 4/5 weakness of the iliopsoas, quadriceps and hamstring muscles of the lower limbs. Feet and toe extensors showed severe 2/5 weakness. In both patients there was mild weakness of the adductors and the calf muscles and the glutei were normal. Knee reflexes were absent, while all other tendon reflexes were normal. In both individuals, distal upper limb muscles showed normal strength. All sensory modalities were intact, as were cranial nerves and there was no ptosis or wasting of the temporal muscles. EMG of both patient 5 (at age 50 years) and patient 6 (at age 28 years) showed no spontaneous activity. Motor unit potentials were small with early recruitment and low envelope curve, signifying myopathic changes.

Family B: The 55 year old mother (individual 4, family B, Fig. 1a), and the affected children (B1, age 18 and B3 age 31 years) showed similar clinical features: weak neck flexors, with contracture of the neck and inability to passively flex the neck. There was very mild proximal weakness of the upper limbs muscles, and moderate weakness of the finger extensors and intrinsic hand muscles accompanied by wasting. There were no contractures of the elbows or the fingers. In the lower limbs there was 4/5 weakness of the iliopsoas and feet and toes extensors with contractures of Achilles tendons. The extensor digitorum brevis muscles were hypertrophied. All tendon reflexes were weak, but elicitable. Patient B3 showed also mild winging of the scapulae and patient B1 had severe scoliosis.

Age of onset of initial symptoms varied between patients: In family A (Fig. 1A), while patient 6 was symptomatic already at 2 years of age, patients 1, 5 and 7 recall initial symptoms at ages 30, 14 and 18 years, respectively. In family B, all affected individuals are documented to have been symptomatic within the first year of life. Creatine phosphokinase levels were elevated in most affected individuals of both families (range 86-682 IU/L), and were normal in unaffected family members tested (including individual 3, family A, age 40, carrying the mutation). Echocardiography was normal for all affected individuals of both families.

Muscle biopsies from the vastus lateralis were obtained for one affected individual of each family, processed through various staining procedures as described in Materials and Methods, demonstrating non-specific changes. In patient 5, family A, a muscle biopsy done at the age of 50 years showed remarkable variability in fiber size with numerous internal nuclei. Many fibers showed longitudinal splitting. There were a few ring fibers and many tiny fibers were dispersed throughout, with a mild increase in subsarcolemmic glycogen. Most of the fibers were Type 2. All the immunocytological reactions and stains were normal. The interpretation of these findings was nonspecific myopathic changes. The muscle biopsy of patient 4, family B, demonstrated well preserved general structure, with random variability in fiber sizes with no atrophic fibers. Most of the fibers were Type 2. The intramyofibrillary network was intact and there was no accumulation of glycogen or fat. Thus, the changes were mild and nonspecific.

### Magnetic resonance imaging (MRI)

**Fig. 2:**
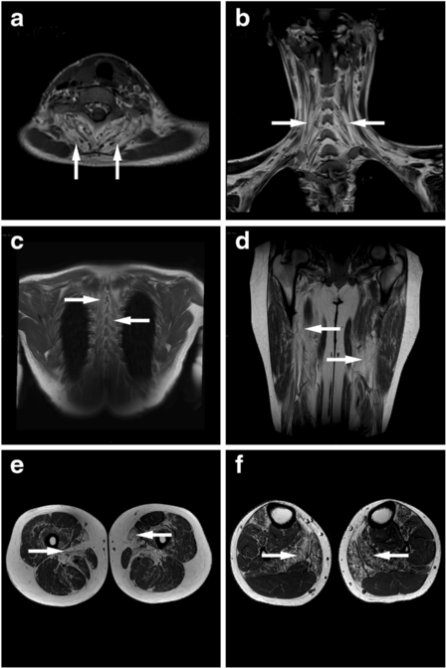
Muscle imaging (MRI) of affected individual 6, family A, 28 year old male. **a** Axial T1WI of neck demonstrating selective fibro-fatty degeneration of the extensor muscles. **b** Coronal T1WI of neck showing defuse fibro-fatty degeneration of the extensor muscle extending to the back. **c** Coronal T1WI of mid back demonstrating defuse fibro-fatty degeneration of the para-spinal muscles. **d** Coronal T1WI of thigh showing prominent fibro-fatty degeneration of the adductor muscles. **e** Axial T1WI of thigh demonstrating prominent fibro-fatty degeneration of the adductor muscles. **f** Axial T1WI of calf showing selective fibro-fatty degeneration of soleus muscle. Arrows highlight loci of fibro-fatty degeneration

Cross sectional MRI provides a non-invasive high resolution tool evaluating muscle physiopathology and morphology in neuromuscular disease. Muscle imaging (MRI) carried out on affected individual 6, family A (28 year old male) showed fibro-fatty degeneration noted as hyperintense confluent replacement in extensor muscles of the neck (Fig. 2a,b) and back (Fig. 2c). Extensive involvement was noted also in the lower limbs, specifically in the tibialis anterior, soleus and gastrocnemius medialis at lower leg and mostly adductors and sartorius at thigh level (Fig. 2d,e,f).

### Identification of the MYH7 mutations

Considering the phenotypic similarity observed in families A and B, we sought for a common genetic defect in both families. Whole exome sequencing (WES) of individual 6 of family A (Fig. 1a) was the initial molecular study done, revealing 8 polymorphisms in genes responsible for Limb-Girdle Muscular dystrophy (LGMD) that was suggested as an initial diagnosis. All variants in the genes *TTN* (LGMD2J, MIM#608807), *DYSF* (LGMD2B, MIM#253601), *TCAP* (LGMD2G, MIM#601954), *SGCA* (LGMD2D, MIM#608099) and *PLEC1* (LGMD2Q, MIM#613723) found by WES analysis, were ruled out by Sanger sequencing as they were not shared by all affected family members of family A (data not shown). Several other variants in these genes and others, previously connected to LGMD, were also excluded based on their presence in the NCBI-dbSNP database.

WES was then carried out for individual 9 of family A and individual 3 of family B. Combined analysis of the WES results of individuals 6 and 9 of family A (Fig. 1a) identified 37 heterozygous polymorphisms. However, apart from the c.4309G>C nucleotide change found in *MYH7*, none of those variants was located in myopathy-related genes or was common to the affected individuals in family A (data not shown). Only two of those variants were also present in WES data of individual 3 of family B. However, they are likely not related to the phenotype observed in families A and B, since both variants were found in EVS and NCBI-dbSNP databases.

Sanger sequencing and restriction analysis demonstrated the presence of the heterozygous *MYH7* c.4309G>C variant in all 5 affected individuals of family A, but not in the healthy family members 2 and 8 or in any of 160 control samples of Jewish Moroccan ancestry. However, the mutation was found in one apparently healthy 40 year old female family member (individual 3, family A). In this individual, both careful physical examination (including walking on heels as well as strength of hallucis longus and tibialis anterior) and blood creatine phosphokinase values were normal. Regretfully, this individual was not available for MRI studies, so that the presence of a subtle subclinical muscle phenotype cannot be ruled out. In line with the history of both families, the *MYH7* c.4309G>C mutation was not found in the affected members of family B suggesting that although both were of Jewish Moroccan ancestry, they were not directly related. Interestingly, WES of affected individual 3 of family B, followed by Sanger sequencing of *MYH7*, revealed a c.4301G>C mutation (Fig. 1b), situated only 8 nucleotides upstream to the c.4309G>C mutation in family A. Restriction analysis demonstrated that the c.4301G>C mutation segregated as expected within the affected family B and was not found in the 160 matched controls.

### In vitro studies of functional consequences of the MYH7 mutations

**Fig. 3:**
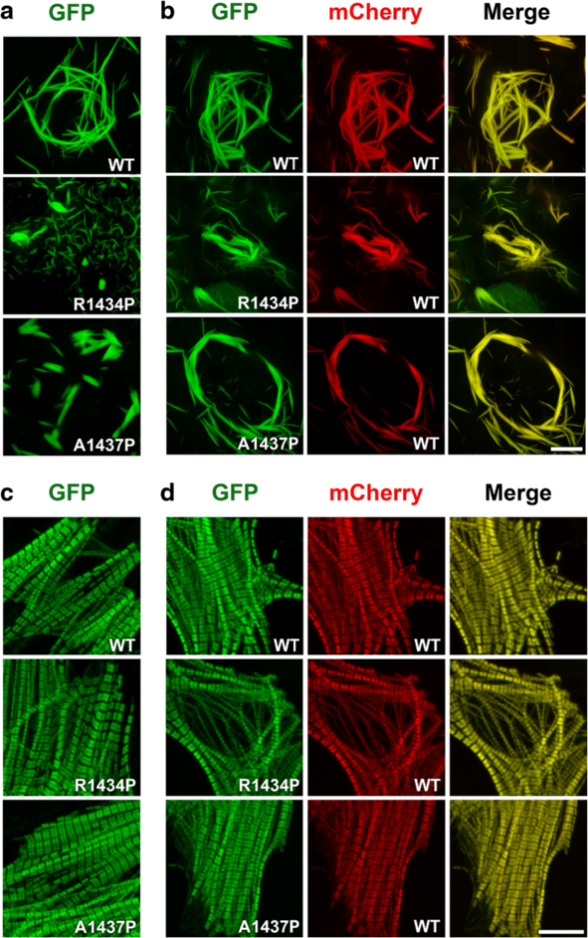
**a**, **b** The two mutations R1434P and A1437P impair myosin self-assembly properties and have dominant negative activity in non-muscle cells. COS-7 cells were transfected with WT and mutant GFP-tagged myosins as reported and imaged by confocal microscopy 12 h later. **a** (GFP): cells transfected with only GFP-tagged myosins. **b** (GFP, mCherry, Merge): cells co-transfected with WT or mutant GFP-tagged and WT mCherry-tagged myosins. Bar corresponds to 10 μm. **c**, **d** The two mutations R1434P and A1437P are efficiently incorporated in cardiomyocytes. NRVMs were transfected with the Amaxa Nucleofector™ Technology and imaged 96 h later by confocal microscopy. **c** (GFP): cells transfected with GFP-tagged myosins using 2 μg of plasmid DNA. **d** (GFP, mCherry, Merge): cells co-transfected with GFP-tagged and WT mCherry myosins using 1 μg of plasmid DNA per construct. Bar corresponds to 10 μm

The families analyzed in this study show phenotypic similarity to MPD1, a progressive distal myopathy caused by MYH7 rod mutations. However, the cervical contracture seen in patients of both families, combined with the absence of clinical phenotype in one individual of family A, raised the question whether the *MYH7* mutations found have any effect on myosin thick filament formation and are the cause of disease. Since proline mutations in the MYH7 rod are very common in patients with MPD1 and their phenotype in non-muscle as well as muscle cells has been previously characterized [11], we tested the effect of the two missense mutations found in our patients (c.4309G>C - p.A1437P; c.4301G>C - p.R1434P). When expressed in non-muscle cells that do not contain other sarcomeric proteins, myosins have the ability to self-assemble into ordered packed filaments that share some of the structural characteristics of the thick filament [12, 13]. To determine whether the A1437P and R1434P substitutions affect myosin assembly properties, we first transfected non-muscle COS-7 cells with β-myosin constructs carrying the R1434P or A1437P substitutions. To visualize the assembly of mutant and WT myosins, the full-length β-myosin molecule was tagged at the N-terminus with the fluorescent reporter gene encoding green fluorescence protein (GFP), and the formation of myosin macromolecular assemblies was followed by confocal microscopy in live cells. We first imaged the GFP-tagged WT and mutant constructs alone, to determine whether the two-proline mutations cause a structural deformation of the rod that negatively impacts myosin self-assembly. When cells were transfected with the WT GFP-tagged myosin construct, we observed the typical long myosin filaments organized in cytoplasmic nest-like structures (Fig. 3a, WT). In contrast, neither mutant self-assembled properly: while the R1434P mutation forms shorter and thinner filaments, the A1437P mutation forms shorter but thicker filaments (Fig. 3a, R1434P, A1437P). Thus, both proline substitutions appear to impact the intrinsic ability of the β-myosin rod to assemble into an organized structure competent for correct filament formation in non-myogenic cell lines.

Since most β-myosin mutations cause disease in an autosomal dominant fashion, in order to mimic the cellular setting occurring in the heterozygous patients, we co-transfected each individual GFP-tagged mutant with mCherry-tagged WT myosin. We monitored changes in the assembly behavior of both alleles. Imaging of cells expressing the two WT constructs revealed co-localization of the two myosins and no apparent changes in the arrangement of the filaments (Fig. 3b, WT). In contrast, co-expression of the WT and mutant myosins had a reciprocal effect on their assembly: while the presence of each mutant reduced the assembly of the WT filaments, their respective molecular organization was partially rescued by the WT myosin which appears to reorganize them in longer filaments (Fig. 3b, R1434P, A1437P). Thus, in spite of their self-assembly-defective phenotype, both mutants can interact with the WT myosin but they handicap its normal filament formation ability.

To study the effect of the two proline mutations in a more physiological environment, we next transfected neonatal rat ventricular myocytes and examined whether the mutant myosins are competent for incorporation into the sarcomere. The ordered architecture of this cellular platform provides the resolution necessary to study pathological changes in myosin assembly properties. When compared with the WT construct, both mutations showed normal incorporation and distribution along the A-band of the sarcomere that corresponds to the thick filament (Fig. 3c, compare WT, R1434P and A1437P). Furthermore, no myosin aggregates were detected in the cytoplasm of the cells. Finally, as inferred by the merged panels, the GFP tagged mutants also perfectly co-localized with the mCherry-tagged WT myosin (Fig. 3d). Therefore, as previously reported for other proline substitutions causing MPD1 [11], the two mutations analyzed in this study do not appreciably impair mutant incorporation into the sarcomere in spite of the fact that i) they are predicted to distort the coiled-coil structure of the myosin rod and ii) they have self-assembly defects in non-muscle cells.

## Discussion

Our study identified two novel mutations in the *MYH7* rod domain, and expands the clinical spectrum of skeletal myopathies associated with *MYH7* mutations. Affected individuals have weakness in both proximal and distal muscles in the upper and lower extremities, with invariably severe inability to flex the neck. Contractures were mostly confined to the neck muscles in patients 5 and 6 (family A), and patients 1,3 and 4 (family B). Patients in family A also had various degrees of contracture of the Achilles tendon causing limited dorsiflexion, and patient 6 of family A had also mild elbow contracture. Interestingly, MRI studies of patient 6 of family A revealed diffuse fibro-fatty degeneration in the extensor muscles of the neck and back with extensive involvement also in the lower limbs, mainly in adductors muscles of the thigh and the posterior compartment of the calf. These changes are apparently the cause of the aberrant rigidity seen in the affected individuals. Muscle contractures have been reported in early-onset *MYH2*-associated myopathy and in embryonic myosin heavy chain mutations associated with arthrogryposis [14]. However, they are unusual in MYH7 myopathies with late onset. Thus, *MYH7* mutations should be sought in patients with severe cervical muscle contractures. As previously reported for other MPD1 proline mutations, the two novel mutations presented here do not cause apparent cardiac phenotypes, despite the fact that β-myosin is expressed in both cardiac and skeletal muscle.

Both proline substitutions characterized in this study have similar cellular phenotypes. However, while the initial symptoms in most cases of family 1 were evident only after the age of 15 years, in family B, all affected individuals were symptomatic by the age of 1-2 years. Remarkably, one mutation carrier (individual 3 of family A, age 40) represents a case of practical non-penetrance since we did not find any evident clinical phenotype. Nevertheless, this finding is in line with previous MDP1 studies showing late onset presentations [7]. Thus, partial penetrance of *MYH7* mutations should be discussed in genetic counseling of affected families.

The structural unit of the myosin rod corresponds to a 28-amino acid repeating unit, which contains the classical coiled-coil heptad repeat consisting of a pattern of seven amino-acids. The heptad repeat positions are labeled *a, b, c, d, e, f, g*: hydrophobic residues are generally located in *a* and *d* positions whereas positions *e* and *g* are generally occupied by polar or charged residues. To date, about 350 disease-causing mutations have been found in the β-myosin gene; more than 130 of them are located in rod domain of the molecule (http://bmf2.colorado.edu/myomapr/index.psp). The two mutations characterized in this study, p.R1434P and p.A1437P, introduce a proline in the heptad positions *c* and *f* respectively, and replace amino acids that are highly conserved. Since proline residues cannot act as hydrogen bond donors, their presence in the middle of an α-helical strand causes a kink in the axis of the α-helix of approximately 26° [15]. Thus, proline mutations mapped in the myosin rod are predicted to introduce local conformational changes that could also trigger coiled-coil instability. As previously proposed for MPD1 proline mutations [11], these two structural conditions can induce the formation of protein cytoplasmic aggregates by interfering with the formation of proper myosin-myosin interactions. However, the data presented here indicate that in spite of their defective self-assembly, the two mutant myosins do not aggregate in muscle cells. Thus, it is tantalizing to speculate that, as suggested by the COS-7 cell experiments, the presence of WT myosin could clear the mutant from the cytoplasm before aggregation occurs by assisting its incorporation into the sarcomere. In this regard, we have previously shown that WT/mutant heterodimeric myosin molecules are assembled and incorporated into the thick filaments [11]. Consequently, we believe that, based on their normal incorporation in the thick filaments, the two mutations analyzed here probably exert their dominant negative activity directly on sarcomere mechanics. Specifically, the destabilization of the rod caused by the structural changes introduced by both prolines could affect force generation/transmission as well as enhance the turnover of the thick filament. Since β-myosin is also the main isoform expressed in human heart, it is surprising that the two proline mutations analyzed in this study, as well as many of those associated with MPD1 [16], do not elicit a detectable cardiac phenotype. Remarkably, the majority of non-proline substitutions mapped in the myosin rod cause hypertrophic or dilated cardiomyopathy and only a very small subset of them located at the C-terminus of the rod also has pathological effects on skeletal muscle fibers [16]. Thus, the two molecular settings that operate in the heart and in skeletal muscle modulate the dominant negative activity of β-myosin rod mutants by responding in a different manner to the presence of proline versus non-proline rod substitutions. In addition, both the tissue-specific differences discussed above, as well as the diverse distal muscle phenotypes that are associated with β-myosin proline mutations [16–19], and the phenotype described in this study are probably controlled by the activity of genetic modifiers that can impact the development and severity of the disease by controlling the activation or repression of cellular pathways involved in muscle physiology [20]. In summary, our study reports a partially penetrant phenotype characterized by neck extensor contractures that is caused by two adjacent proline substitution mutations in *MYH7* (p.A1437P, p.R1434P), and demonstrates the importance of functional studies in proving mutation pathogenicity, enabling proper genetic counseling.

## Conclusions

This study expands our clinical and molecular knowledge of *MYH7* rod mutations causing skeletal myopathies, and highlights the importance of discussing disease penetrance during genetic counseling.

## Abbreviations

dbSNP, database of single nucleotide polymorphisms; GFP, green fluorescence protein; LDM / MPD1, Laing distal myopathy; LGMD, limb girdle muscular dystrophy; MRI, magnetic resonance imaging; MyHC, myosin heavy chain; TR, repetition time; WES, whole exome sequencing; WT, wildtype
